# Hispanic worker attitudes toward pig euthanasia on U.S. farms

**DOI:** 10.3389/fvets.2024.1281102

**Published:** 2024-04-02

**Authors:** Nancy F. Acevedo León, Pablo Lamino Jaramillo, Carlos Durán Gabela, Amy Boren-Alpízar, Allison Andrukonis, Marcelo Schmidt, John McGlone, Arlene Garcia

**Affiliations:** ^1^School of Veterinary Medicine, Texas Tech University, Amarillo, TX, United States; ^2^Animal and Food Sciences Department, Texas Tech University, Lubbock, TX, United States; ^3^Department of Agricultural Education and Communication, University of Florida, Gainesville, TX, United States; ^4^Agricultural Education and Communications Department, Texas Tech University, Lubbock, TX, United States; ^5^School of Animal Sciences, Virginia Tech, Blacksburg, VA, United States

**Keywords:** Common Swine Industry Audit (CSIA), North Ameria Free Trade Act (NAFTA), hispanic workforce, moral injury, secondary traumatic stress

## Abstract

**Introduction:**

On-farm pig euthanasia considers aspects of animal welfare and industry economics. Guidelines are available about the euthanasia process, but the agricultural workforce is highly diverse and guidelines do not consider cultural barriers. Euthanasia requires the ability to identify compromised pigs, technical skills, and willingness to euthanize pigs. In addition, timely euthanasia is part of the Common Swine Industry Audit (CSIA) and, thus, can lead to failed audits if not performed as required by the audit standards. The United States (US) swine industry employs a high percentage of Latin American workers, some US residents/citizens, and others through non-immigrant North American Free Trade Agreement (NAFTA) visas. These workers vary in their level of education and swine industry experience. Proper training of this workforce and identification of the barriers associated with performing timely euthanasia are critical to promote improved welfare practices. The objectives of this study were to: (1) develop a survey instrument to identify Hispanic caretaker attitudes toward pig euthanasia, (2) assess and describe swine caretakers’ attitudes toward pig euthanasia using the developed survey instrument, and (3) determine the demographic and psychological barriers associated with performing pig euthanasia.

**Methods:**

Participants (*n* = 163) were surveyed from 16 farms across the State of Iowa. The on-farm survey was administered for two days in a period of 60 min per day.

**Results:**

The results for demographics and the swine management survey data indicated that employees with less time working on the farm showed less knowledge of the CSIA, lower perceived ability to identify compromised pigs that needed to be euthanized, lower willingness to pecrform euthanasia on their own, and preferred not to have the responsibility of telling others when to euthanize pigs (*p* < 0.001). Secondary traumatic stress and transgressions were significantly correlated scales, associated with burnout, betrayals, and worker satisfaction (*p* = 0.022). Furthermore, individuals identifying as female had higher secondary traumatic stress scores (*p* = 0.026) and lower compassion satisfaction scores (*p* = 0.015).

**Discussion:**

This data suggest that there are demographic, psychometric, and training-related factors correlated with Hispanic caretakers’ feelings about pig euthanasia. The results of this study could be used to further improve and develop targeted training programs for Hispanic caretakers for early identification of compromised pigs and timely euthanasia, which could benefit human well-being, animal welfare, and the swine industry audit performance.

## 1 Introduction

According to the National Pork Board [NPB], euthanasia is defined as the humane process, whereby the pig is rendered unconscious, with minimal pain and distress, until death. The word “euthanasia” is derived from the Greek terms “eu,” meaning good, and “Thanatos” meaning death, defining euthanasia as good death (1–3). Euthanasia decisions for animals exhibiting symptoms of disease or distress (referred to as compromised pigs) should be made as early as possible and should be based on the severity of the animal’s condition, previous treatment knowledge, observation, and transport for slaughter or removal to another location for diagnostic purposes (4–7).

To determine if a method of euthanasia is humane, some key elements include whether an animal experiences minimal pain and distress, rapid loss of consciousness, and death achieved quickly and consistently (3). Animal welfare requires disease prevention and veterinary treatment, appropriate shelter, management, nutrition, humane handling, and humane euthanasia (8). Euthanasia is necessary when a pig is sick, injured, or in poor condition (3). Workers are required to be trained according to NPB’s Pork Checkoff, Pork Quality Assurance Plus program (9) (PQA Plus 2022; training and certification) before handling/euthanizing any animals. Farm protocols must be objective in their approach to allow caretakers to make accurate, consistent, and timely euthanasia decisions. According to Morrow et al. (10), veterinary decisions for the euthanasia of companion animals follow both subjective and objective guidelines. For example, some subjective measures may involve the ability of the animal to enjoy food, breathe freely and without difficulty, and eat and drink without pain (10). In contrast, objective guidelines evaluate weight loss, weakness, infection, organ failure, and injuries (11).

Performing euthanasia is a multi-step process requiring observational abilities to identify compromised pigs and the technical skills and willingness to humanely euthanize these animals (12). Timely euthanasia in pigs is critical for meeting good production practices and mitigating animal suffering. The swine industry has struggled to identify the barriers associated with timely euthanasia. The reasons for not performing timely euthanasia or not performing it at all may vary. Hartnack et al. (13) reported that veterinarians who were newer to the profession were more likely to disagree with euthanasia in some scenarios.

Not performing euthanasia or delayed euthanasia can lead to animal distress and suffering. However, the swine industry struggles to understand why, after adopting methods to train caretakers through videos, in person training, and yearly trainings, timely euthanasia is still not taking place. Swine caretakers can significantly compromise animal welfare due to delays in pig euthanasia. Matthis et al. (14) conducted a survey determining that ethnic background and gender affect employee attitudes toward euthanasia. Hispanic female employees and employees with certain personality traits had more negative attitudes toward euthanasia. However, all employees preferred a method of euthanasia that is perceived as less painful to a pig, suggesting that the animal’s suffering is concerning to caretakers. A study by McGee et al. (15) revealed that many swine caretakers agree that it is humane to euthanize ill pigs and it is crucial to have good skills for euthanasia.

Identifying exactly what the barriers are in conducting timely euthanasia is complex as it can be multi-factorial. “Compassion fatigue” was first described as distress experienced by those in human nursing positions (16), but it has recently been used to describe the emotional effects of euthanizing animals (17). Additionally, research on animal shelter employee well-being has suggested that moral injury may be correlated with euthanasia (4). It has been suggested that caretakers may experience a wide range of negative emotions, including grief or distress, when performing euthanasia (6). As such, Arluke (18) coined the phrase the “caring-killing paradox” to describe the stress experienced by animal shelter workers tasked with euthanizing the same companion animals they provided care for. Both compassion fatigue and caring-killing paradox have been suggested as factors affecting euthanasia decisions on swine farms (12).

Even with established guidelines in the Common Swine Industry Audit [CSIA], euthanasia decision-making varies among farms. The NPB provides practical recommendations on swine euthanasia in the On-Farm Euthanasia of Swine: Recommendations for the Producer document (3). The CSIA requires that animals with certain conditions can be euthanized (19). These conditions include pigs that have been treated for 2 consecutive days without showing improvement, pigs with perforated or large enough hernias that cause ulcerations and make it difficult for them to walk, pigs with uterine or rectal prolapses exhibiting signs of necrosis, severely injured, or non-ambulatory pigs that are unable to recover, and non-ambulatory pigs with a body condition score of one. Although the guidelines are clear, workers struggle to make timely decisions followed by euthanasia. One speculation is that maybe workers do not feel confident in identifying certain conditions and may feel like they are making the wrong decision in conducting euthanasia.

Identification of compromised pigs is critical for prompt treatment or timely euthanasia. Compromised pigs are often moved to hospital pens. Hospital pens are designed to house ill animals being treated and nursed back to health. However, most hospital pens function as “sick pens” where animals are not commonly treated (7). Properly operated hospital pens (those offer extra warmth, good footing, easy access to feed and water, and have established protocols for treatment, culling, and euthanasia) provide an economic return, improve worker morale, and improve the welfare of compromised pigs (7). When a pig dies in a hospital pen, it should always be a surprise. If workers say to themselves that they are happy because a particular pig finally died, euthanasia decision rules are inadequate, and the caretakers have neglected a part of their responsibilities (7). However, this is a common occurrence on many farms. Pigs are neither promptly identified as compromised, treated, nor euthanized, as farms still fail the CSIA due to failure in providing timely euthanasia. The question remains, what is lacking or what are we failing to observe or understand?

In 2022, the United States Department of Agriculture (20) reported 13,222,900 head of pig deaths/losses. However, the exact number associated with on-farm euthanasia performed by workers is neither known nor what number of death/euthanasia practices were associated with COVID-19. We do know that performing euthanasia impacts workers negatively, regardless of whether it was associated with compromised animals euthanized on-farm or market disruptions due to COVID-19.

According to the USDA Economic Research Service in 2021, 21.1 million of US jobs were related to the agricultural and food sectors. In 2014, Latinos/Hispanics accounted for 16.1% of the 146.3 million people employed in the United States (21). According to the U.S. Census Bureau, “the terms “Hispanic,” “Latino,” and “Spanish” are used interchangeably. Some individuals identify with all three terms while others may identify with only one of these three specific terms. People who identify with the terms “Hispanic,” “Latino,” or “Spanish” are those who classify themselves in one of the specific Hispanic, Latino, or Spanish categories (“Mexican, Mexican Am., or Chicano,” “Puerto Rican,” or “Cuban”) and those who indicate that they are ‘another Hispanic, Latino, or Spanish origin.’ People who do not identify with one of the specific origins but indicate that they are “another Hispanic, Latino, or Spanish origin” are those whose origins are from Spain, the Spanish-speaking countries of Central or South America, or another Spanish culture or origin. Origin can be viewed as the heritage, nationality group, lineage, or country of birth of the person or the person’s parents or ancestors before their arrival in the United States. More broadly defined, the term “Hispanic” relates to “a person of Latin American decent that lives in the US” (1).

Approximately 44% of farming, fishing, and forestry workers were Latino/Hispanic (21). Given that over 500,000 agricultural workers are identified as first-generation immigrants of Latino/Hispanic background (22), we hypothesize that cultural differences in caregivers’ attitudes, education level, values, and beliefs may be impacting decisions in timely euthanasia. The North American Free Trade [NAFTA] was established on 1 January 1994 (23). According to the U.S. Citizen and Immigration Services, this agreement created unique economic and trade relationships for the United States, Canada, and Mexico. The Trade NAFTA [TN] (a non-immigrant status classification) allows qualified Canadian and Mexican citizens to pursue temporary entry into the United States to engage in business activities at a professional level, which supports nearly 5 million American jobs. To apply for this agreement, the participant’s profession must meet the regulations, and the applicant must have the qualifications and knowledge required for employment (24). These requirements and regulations are important in the present study, as many of the workers with TN visas classify as agricultural, veterinary, or animal care professionals. It is clear that the Hispanic agricultural workforce is now more diverse (in country of origin and education status), and therefore, understanding the workforce is critical to be able to find solutions and improve animal welfare overall.

There is a gap in the literature identifying attitudes that might affect Hispanic swine caretakers’ ability to perform timely euthanasia. Therefore, the overall goal of this study was to improve animal welfare by identifying the financial, educational, psychological, and cultural barriers in Hispanic swine caretakers that lead to a delay in performing timely euthanasia on-farm and quantify the possible psychological impact on Hispanic caretakers when performing euthanasia. To attain this goal, we pursued the following objectives: (1) to develop an instrument to identify swine caretakers’ attitudes toward pig euthanasia, (2) to assess and describe swine caretakers’ attitudes toward pig euthanasia using the developed survey instrument, and (3) to determine the demographic and psychological barriers associated with performing pig euthanasia.

## 2 Materials and methods

The Human Research Protection Program (IRB2019-225) at Texas Tech University approved this study. Before data collection, the survey was revised by a panel of animal science professionals to ensure that the questions were clear and relevant to the topics. Panel members were asked to provide general feedback and specific feedback on (a) each item’s correspondence with research aims and questions, (b) phrasing of items, (c) sequencing of items, and (d) survey length and readability.

Before beginning the study, the investigation group read and reviewed the survey to familiarize themselves with it and ensure consistency during the research presentation. Data were collected from 16 swine farms located in Iowa. The group size of caretakers vaired among farms, depending on the individual farm’s size, ranging from 7 to 18 members. The survey was conducted exclusively in Spanish since over 90% of the caretakers primarily spoke Spanish. Consequently, English-speaking caretakers were not included in the study due to time limitations. Participants were given clear information about the study’s purpose, confidentiality of their responses, risks involved, and their right to withdraw from the study at any point of time. It is important to note that participation in the survey was entirely voluntary, and all Spanish-speaking caretakers were invited to take part. Lunch was provided, even for those who chose not to participate. For the purpose of anonymity, participants marked their survey with an ID number based on their assigned seats. This ensured the confidentiality of their responses while allowing them to identify their survey on subsequent days, as the study involved two survey sessions. The farms were asked to remove recording equipment to keep the data collection confidential. Researchers started the interaction by introducing themselves. The survey was given for 2 days, and each day, a period of 60 min was used for data collection during the lunch hour/lunch time. Demographic questions, Swine Management, and Impact of Event Scale-Revised (IES-R) were covered on the first day. The Professional Quality of Life and The Moral Injury Event Scales were given on the second day. During the survey, researchers read each question aloud in Spanish, ensuring every participant understood what was asked. Participants were given 1 min to answer each question and extra time for any clarification. Participants were asked to take the same seats starting the second day to get their survey. Again, the protocol was the same as the first day. At the end of the second day, all the surveys were collected and kept in an envelope labeled with the farm identification number.

### 2.1 Swine caretakers’ attitudes toward pig euthanasia survey

, An 83-question survey was used to explore Hispanic caretakers’ attitudes toward pig euthanasia. It consisted of five survey sections: (1) Demographics, (2) Swine Management, (3) The Impact of Event Scale-Revised (IES-R; 25), (4) The Professional Quality of Life Scale (26), and (5) The Moral Injury Event Scale (MIES; 27). The survey sections were used to identify the barriers associated with performing pig euthanasia.

#### 2.1.1 Instrument surveys

##### 2.1.1.1 General demographics

The first section on demographic information incorporated nine questions about gender, age, country of origin, income, education level, education major, time working in the swine industry, time working on the farm, and farm work unit. Demographics establish a profile of the participants and their background, which is crucial for understanding how their experiences and attitudes vary based on these factors.

##### 2.1.1.2 Swine management survey

A group of researchers, with years of expertise in swine management, developed this 19-item dichotomous (yes/no) questionnaire to assess swine caretakers’ attitudes, behaviors, experiences, feelings, and knowledge toward pig euthanasia. This survey directly addresses the experiences and attitudes of swine caretakers in their role, specifically regarding euthanasia. The survey provides insights into worker-related experiences and how these may influence their well-being and relationships with animals.

##### 2.1.1.3 The impact of event scale-revised (IES-R)

This survey section focuses on intrusion assessment, unwanted thoughts, images, dreams, waves of feelings, and repetitive behavior related to the stressor. Avoidance evaluates numb sensation, behavioral inhibition, and awareness of emotional indifference (25). The scale was composed of intrusion and avoidance. The scale ranged from 1 (never) to 6 (very often).

The third section was originally designed to directly measure veterans experienced trauma (28). A previous study by Bride et al. (25) provided a summary of the most utilized instruments using the IES-R for measuring different aspects of compassion fatigue, each reviewed instrument has varying levels of evidence regarding its psychometric properties, and each reviewed instrument was useful for specific purposes, showing a good internal consistency (α = 0.80; 25). The IES-R has been used with animal care employees to evaluate the impact of caring for and killing the same animals (4, 29) showed high internal consistency with Cronbach’s alpha of 0.86.

This scale was used to gauge the psychological well-being of swine caretakers and whether they experience symptoms related to work stress, such as euthanizing pigs. It provides valuable insights into their mental health and potential trauma related to their job.

##### 2.1.1.4 The professional quality of life scale

The ProQOLS consisted of 30 questions. It comprises compassion satisfaction, burnout, and secondary traumatic stress represented in three survey subscales, each including 10 questions. The response options ranged from 1 (never) to 5 (very often). Compassion Satisfaction refers to the satisfaction felt from being able to perform their work efficiently. Burnout refers to feelings of hopelessness and difficulty managing or performing their work correctly. Secondary traumatic stress occurs after an indirect exposure to a traumatic event.

The original validation study indicated that burnout, compassion satisfaction, and secondary traumatic stress have all been found to have acceptable internal consistency with Cronbach’s alpha values of 0.75, 0.88, and 0.81, respectively (26).

This scale provided insights into the emotional well-being of swine caretakers, including their satisfaction with their work, burnout levels, and the impact of direct and indirect exposure to traumatic events. The scale also helped identify factors that may affect social personal ethical convictions of workers.

##### 2.1.1.5 The moral injury event scale (MIES)

The fifth survey section included transgressions and betrayal. The response options ranged from 1 (strongly agree) to 6 (strongly disagree). This instrument has been used previously in a military context (27). Transgression was identified as an act against their ethics or beliefs and betrayal referred to violation of a person’s trust or confidence. This scale has also been used for animal care employees (29).

This scale has shown good internal inter-item consistency (α =0.86) for the full 11-item scale and an excellent (α =0.90) inter-item internal consistency for the shortened 9-item scale (27).

A 9-item shortened scale by Nash et al. (27) was used in this study; the fifth question (“I violated my morals by failing to do something I felt I should have done”) was removed from the analysis to adapt the scale to our study goals and fit with the time provided to complete the survey. To guarantee that the three scales were not affected by the modification, a confirmatory factor analysis (CFA)was conducted. The CFA showed that the deletion did not affect the model fit indices, CFI = 0.90, χ^2^ = 628.98, df = 28, *p* < 0.001, SRMR = 0.061. Additionally, the inter-item consistency was measured for the two scales, showing an acceptable inter-item consistency (α = 0.73) for betrayals’ construct and a good inter-item consistency for transgression (α = 0.86).

Since the target population was Hispanic, the Spanish version of the ProQOLS and Impact of Event Scale-Revised (IES-R) were used. Additionally, MIES, demographic, and swine management questions were translated into Spanish. In addition, the researchers translated MIES, demographic questions, and swine management questions to Spanish, to ensure that Hispanic participants could fully comprehend, respond to, and be unhindered by language barriers.

This scale was used to explore the moral and ethical aspects of swine caretakers’ work, focusing on perceived violations of their values. It also elucidated how their work may impact their ethical principles, potentially affecting their social relationships and well-being.

Collectively, the instruments used provided a comprehensive view of the psychological, emotional, and ethical dimensions of swine minority and Spanish speaking caretakers’ experiences.

#### 2.1.2 Data cleansing

The data were transferred to Excel®. In total, 175 participants filled out the survey. Incomplete responses (less than 50% of the survey) and participants without euthanasia responsibilities were not considered. Then, 12 outliers and extreme values were removed based on Cook’s distance analysis as estimation of the influence of our participants’ data collected (30). After data cleansing, the final number of valid responses was 163 (93.14%).

#### 2.1.3 Data analysis

Data were analyzed using Statistical Package for Social Sciences (SPSS) v.27 and Statistical Analysis System (SAS) v.9.4. An alpha level of 0.05 was established as *a priori* for all inferential analyses. Descriptive statistics were used to characterize participants’ demographic information. The chi-square test was run to determine caretakers’ characteristics that influenced performing euthanasia. The instrument was revised to make specific statements about helping animals.

Mann–Whitney U and Kruskal–Wallis tests were used to determine the median differences between groups of demographic variables in the psychological scales. The demographic variables included gender, age, country of origin, income, education level, education major, time working in the swine industry, time working on the farm, and work unit. The outcome variables included compassion satisfaction, burnout, stress, transgression, and betrayal.

Spearman’s rank-order correlations were used to determine the relationships among all the outcome variables. Then, correlations were made between scales to understand how a variable was related to another variable. These analyses helped to find the variation of strength and direction relation and predicted a construct based on another one.

## 3 Results

The first objective of the study was to create a tool that would help in assessing the attitudes of swine caretakers toward pig euthanasia. To accomplish this objective, the researchers adapted and utilized three scales, namely, The Impact of Event Scale-Revised (IES-R), The Professional Quality of Life Scale (ProQOLS), and The Moral Injury Event Scale (MIES). After conducting the necessary analysis, the following are the key findings for objective one.

### 3.1 The impact of event scale-revised (IES-R)

The IES was not utilized entirely; due to the fact that before the study, some items were reviewed and removed by a panel member justifying, they did not correspond with the research aims and the purpose of an improvement length and readability of the instrument. The results from this scale did not present any variability (*M* = 1, *SD* = 0). Thus, it was removed from further analysis.

### 3.2 The professional quality of life scale (ProQOLS)

In the present study, compassion satisfaction (α = 0.77), burnout (α = 0.70), and secondary traumatic stress had acceptable inter-item consistency (α = 0.71).

### 3.3 The moral injury event scale (MIES)

Based on the previously mentioned item selection variability, researchers adapted it by removing item five (“I violated my morals by failing to do something I felt I should have done”). To guarantee that the three scales were not affected by the modification, confirmatory factor analysis [CFA] was conducted. The CFA showed that the deletion did not affect the model fit indices, CFI = 0.90, chi-square = 628.98, df = 28, *p* < 0.001 SRMR = 0.061. Additionally, both betrayal (α = 0.73) and transgression construct (α = 0.86) showed acceptable reliability.

### 3.4 To assess and describe swine caretakers’ attitudes toward pig euthanasia using the developed survey

The second objective aimed to investigate the attitudes of swine caretakers toward pig euthanasia using the survey developed from the first objective. To achieve this objective, the researchers used the demographic information and the swine management survey to identify characteristics that influence euthanasia.

### 3.5 Demographic survey frequencies

**Figure 1:**
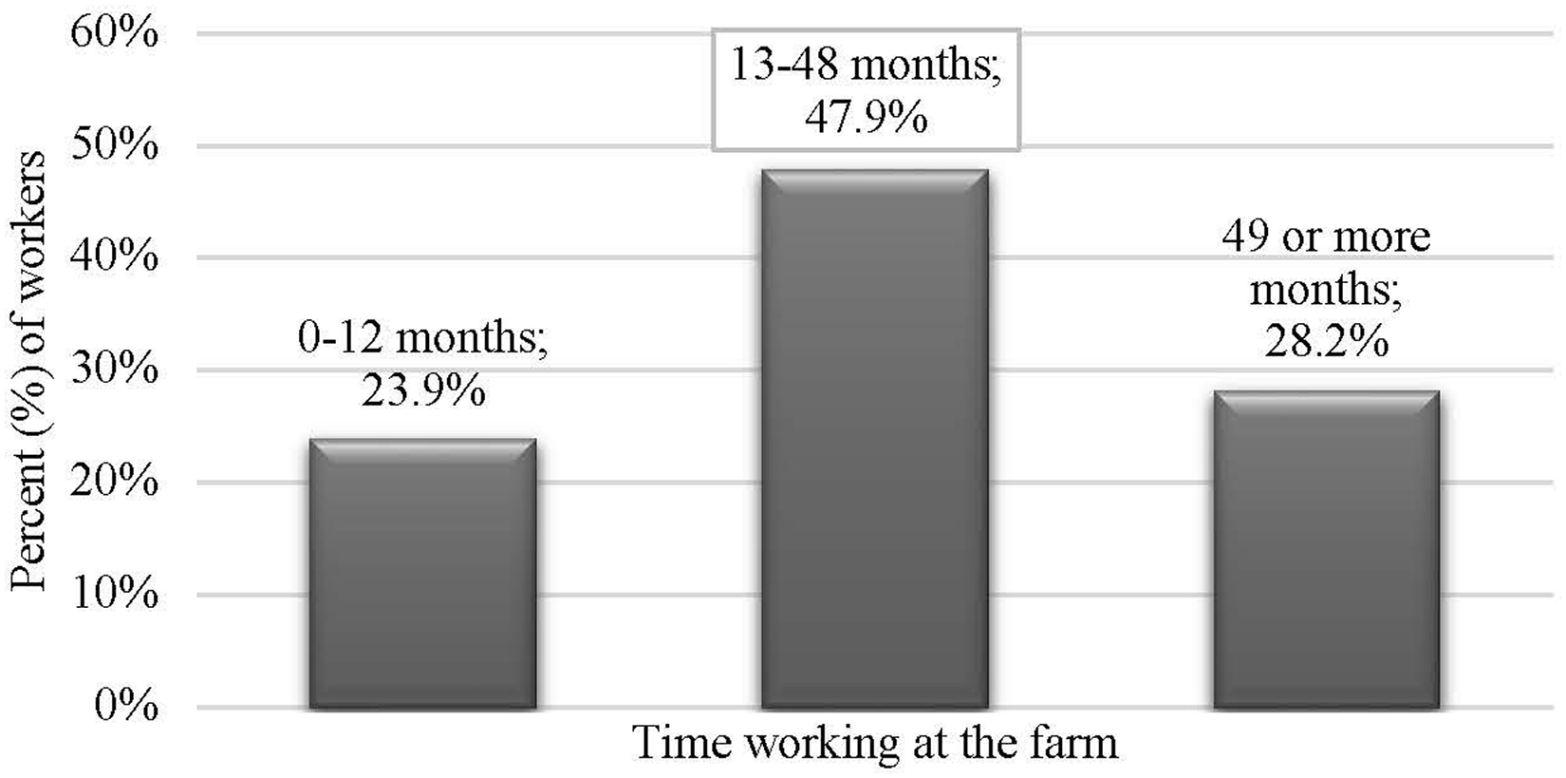
Distribution of participants by time working in the swine industry.

**Figure 2:**
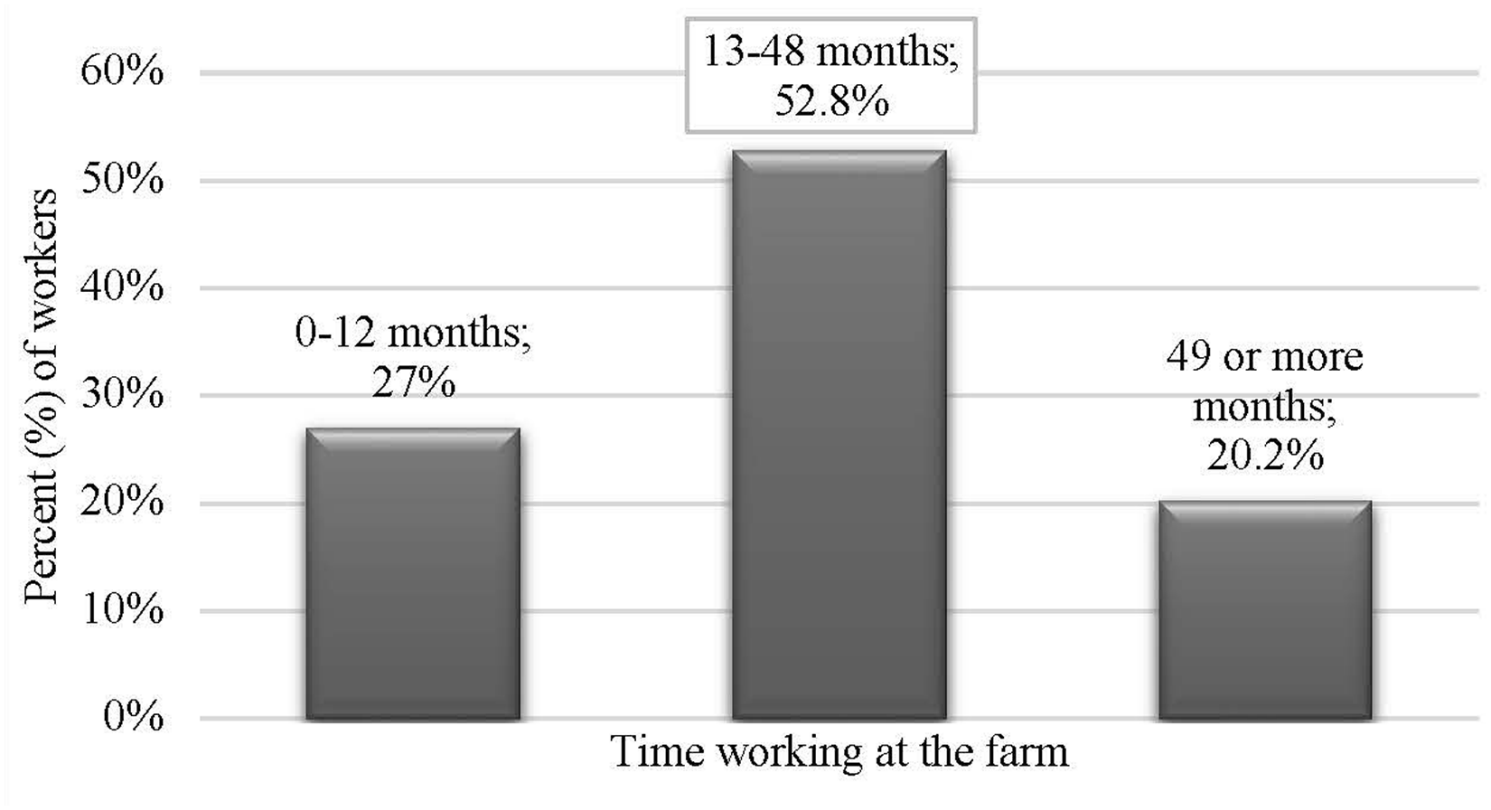
Distribution of participants by time working at the farm.

**Figure 3:**
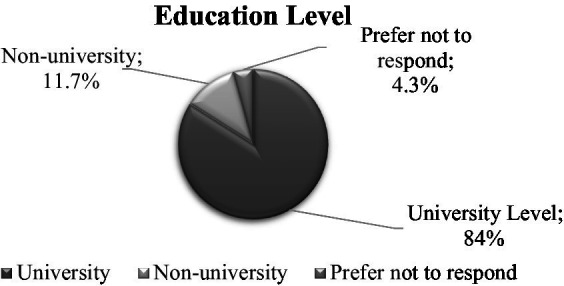
Distribution of participants by *educational level.*

**Figure 4:**
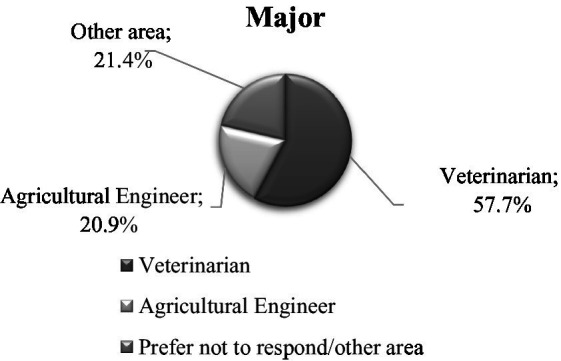
Distribution of participants by education *major.*

**Figure 5:**
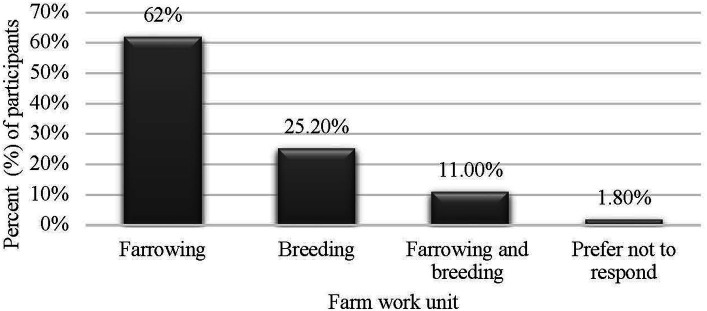
Distribution of participants by farm work unit.

Most of the participants identified as male (*n* = 107; 65.6%), female (*n* = 52; 31.9%), or preferred not to disclose (*n* = 4; 2.5%) and were mainly young adults between 18 and 35 years of age (*n* = 116; 71.2%). Just 6.7% of the participants were older than 46 years of age. Most of the participants were either Mexican (*n* = 156; 96.9%), Mexican-American (*n* = 2; 1.2%), Guatemalan (*n* = 1; 0.6%), or Honduran (*n* = 1; 0.6%). Over a quarter of participants (*n* = 43; 26.4%) earned between 29,000 and 34,000 USD. A majority of participants had a university degree (*n* = 137; 84.0%), with more than half majoring in veterinary medicine or animal husbandry (*n* = 94; 57.7%). Approximately 50% of participants indicated that they had been working in their current farm for 13 to 48 months (approximately 4 years). Finally, 62% of caretakers worked in a farrowing farm unit (*n* = 104), 25.2% worked in a breeding unit (*n* = 41), and 11% worked in both units simultaneously (*n* = 18). Figures 1–5 and Table 1 show demographic information of the participants.

**Table 1:** Frequencies for participant age and income distribution variables.

Characteristic	*n*	%
Age (years)		
18–35	116	71.2
36–45	34	20.9
46-more	11	6.7
Prefer not to respond	2	1.2
Income ($/per year)		
19,000-24,000	30	18.4
24,001-29,000	26	16.0
29,001-34,000	43	26.4
34,001-39,000	25	15.2
39,001-more	26	16.0
Prefer not to respond	13	8.0

According to the demographic survey results, even though euthanasia was a responsibility of all participants in this analysis, almost half (*n* = 69; 42.3%) of the participants did not like to euthanize pigs. All participants dealt with sick or injured pigs and felt that they could differentiate a healthy pig from a compromised pig. Although the majority of caretakers were formally trained to recognize compromised pigs (*n* = 156, 95.7%), some caretakers (2.5%; *n* = 4) did not have time during their shift to identify sick or injured pigs.

A few caretakers (*n* = 9; 5.5%) stated that they wait for another person to identify sick/injured pigs. Most of participants (*n* = 156, 95.7%) believed that the hospital pen benefits compromised pigs. In addition, most participants (*n* = 148, 90.8%) also recorded when moving a pig into a hospital pen. However, a smaller percentage of caretakers (*n* = 135; 82.8%) regularly check these facilities.

Some caretakers (*n* = 129; 79.1%) consider telling others when to euthanize a pig as part of their job. Most caretakers (*n* = 160; 98.2%) know when to perform euthanasia immediately according to the farm action plan, and most participants (*n* = 154, 94.5%) identify and euthanize a pig immediately according to the farm criteria; however, only 117; 71.8% of caretakers decide when an animal needs to be euthanized and perform euthanasia themselves. Moreover, some caretakers (*n* = 32, 19.5%) consider it easier if someone else identifies the sick/injured pigs for them and then they themselves perform euthanasia. On the otherhand, some caretakers, (*n* = 55, 33.7%) prefer to identify the compromised pigs and prefer for someone else to perform euthanasia. Table 2 describes the frequencies of Swine Management section.

**Table 2:** Frequencies for the swine management section.

		Yes		No	
	Item	*n*	%	*n*	%
1	I deal with sick or injured pigs	163	100	0	0
2	I can differentiate a healthy pig from a sick/injured one	163	100	0	0
3	I was formally trained (with videos, by a supervisor or trainer) to identify sick/injured pigs	156	95.7	7	4.3
4	I have enough time in my shift to identify the sick/injured pigs	159	97.5	4	2.5
5	I wait for another person to identify the sick/injured pigs, and did not do it myself	9	5.5	154	94.5
6	I know what a hospital/isolation pen is (hospital pen)	156	95.7	7	4.3
7	I know why pigs are moved to hospital pens.	158	96.9	5	3.1
8	I believe that hospital pens benefit sick/injured pigs.	156	95.7	7	4.3
9	I regularly check the hospital/sick pens.	135	82.8	28	17.2
10	I complete the records when I move pigs to sick/hospital pens.	148	90.8	15	9.2
11	I know what the Common Swine Industry Audit (CSIA) is.	91	55.8	72	44.2
12	Euthanasia of pigs is part of my job.	163	100	0	0
13	Part of my job is telling others when to euthanize a pig.	129	79.1	34	20.9
14	I like to euthanize pigs.	94	57.7	69	42.3
15	I know when a pig must be euthanized immediately according to the Euthanasia plan of my farm.	160	98.2	3	1.8
16	It would be easier for me if someone else identified the sick /injured pigs that need to be euthanized and then I euthanize them.	32	19.6	131	80.4
17	It would be easier for me to identify sick/injured pigs and then someone else euthanized them.	55	33.7	108	66.3
18	If I see a pig that should be euthanized (according to the criteria of the plans of Action / SOP from my farm), I euthanize it immediately	154	94.5	9	5.5
19	I decide when an animal needs to be euthanized and perform the euthanasia by myself.	117	71.8	46	28.2

#### 3.5.1 Caretakers characteristics that influence euthanasia according to demographics and swine management survey

The chi-square test was run to determine the characteristics of swine caretakers, influencing their perception of performing euthanasia. A total of 22 chi-square analyses were performed, reporting significant differences for each demographic variable item compared with the swine management section survey items. Specific characteristics among the caretakers seem to influence practices related to the euthanasia process in the swine industry, such as education level, time working in the swine industry, time working in the farm, income range, and work unit. Gender and age showed no statistical differences in swine management practices.

#### 3.5.2 Common swine industry audit knowledge by swine industry/farm experience and farm work unit

A chi-square test of independence was calculated by comparing the swine management (yes = 1, no = 2) and demographic questions. There was a significant difference between Common Swine Industry Audit (CSIA) knowledge and the time working in the swine industry (*X*^2^ = 2, N = 163, *p* < 0.001). Participants with more than a year of experience in the swine industry were more likely to know about the CSIA, whereas 82.05% (*n* = 32) of caretakers with less than a year of experience did not have knowledge of the CSIA. Additionally, over a quarter of the most experienced (more than 49 months) caretakers are not aware of this audit 28.26% (*n* = 13).

Finally, participants’ responses showed a significant difference in knowledge of the CSIA, depending on what farm unit they worked in (*X*^2^ = 2, *N* = 160, *p* = 0.005). More than half (53%; *n* = 54) of the farrowing caretakers did not have knowledge of the CSIA compared with caretakers in the breeding area (26.83%; *n* = 11) or caretakers assigned to both areas (farrowing and breeding) (27.78%; *n* = 5).

#### 3.5.3 Easier if someone else identifies the compromised pigs by education level and work experience

There was a significant difference in the relationship between caretakers who found it easier if someone else identifies the compromised pigs for them to euthanize (*X*^2^ = 1, *N* = 156, *p* = 0.010). Most caretakers with a university degree (83.21%, *n* = 114) did not consider it easier if someone else identifies the sick/injured pigs for them to euthanize. In comparison, 42.11% (*n* = 11) of those without university education did consider it easier if someone else identifies the compromised pigs for euthanasia.

Assistance in identifying compromised pigs that need to be euthanized also varied by time working at the farm (*X*^2^ = 2, *N* = 163, *p* < 0.001). In total, 31.82% (*n* = 14) of the less experienced caretakers (fewer than 12 months working at the farm) would prefer it if someone else identified the compromised pigs, and then after the identification, the less experienced caretakers would perform euthanasia. Meanwhile, just 18% of the caretakers with more than a year of experience of working at the farm (13–49 months) think it is easier if someone else identifies the compromised or injured pigs that need to be euthanized, and then, after the decision is made by another person, the caretakers with 13–49 months of experience perform the process on their own.

#### 3.5.4 Capacity to identify compromised pigs and perform euthanasia on their own by time working in industry and at the farm

The results between the time working at the farm vary (*X*^2^ = 2, *N* = 163, *p* < 0.001). In total, 52.27% (*n* = 23) of the less experienced caretakers (0–12 months) at the farm do not identify and perform euthanasia themselves, while 18.60% of caretakers with 13–49 months (approximately 4 years) of experience do not feel capable of performing euthanasia. Finally, 21.21% (*n* = 7) of caretakers with more than 49 months (approximately 4 years) of experience at the farm neither feel capable of identifying compromised pigs nor performing euthanasia themselves.

#### 3.5.5 Possible demographic and psychological barriers associated with timely pig euthanasia performance

The third objective of the study was to identify the demographic and psychological factors that act as barriers to timely euthanasia. This objective was accomplished by analyzing the participants’ demographic information based on the adapted categories of the Professional Quality of Life Scale (ProQOLS) and the Moral Injury Event Scale (MIES).

#### 3.5.6 Mann–Whitney U test between two demographic categories and the professional quality of life scale (ProQOLS)

The Mann–Whitney U Test was used to compare differences between demographic questions, which only included two categories: gender and education level groups, which meets the assumptions for this analysis, as one of the requirements of a Mann–Whitney U test is that the independent variable should be two independent and categorical groups in relation to the ProQOLS.

Compassion satisfaction scores for men (Mdn = 4.20, *SD* = 0.51) were higher than women (Mdn = 4.00, *SD* = 0.43), U = 2,120, z = −2.44, *p* = 0.015. Secondary traumatic stress scores for men (Mdn = 1.44, SD = 0.44) were lower than women (Mdn = 1.67, SD = 0.57), U = 2,178, z = −2.23, *p* = 0.026.

Finally, compassion satisfaction scores for caretakers with a university education level (Mdn = 4.10, *SD* = 0.48) were higher than non-university education levels (Mdn = 3.80, *SD* = 0.54), *p* = 0.052, U = 1,595, z = 1.05, *p* = 0.052. Table 3 shows the frequencies and differences of medians for gender and university level in the ProQOLS.

**Table 3:** Frequencies and Means for difference of medians for gender and university level in the Professional Quality of Life scale.

	Stress			Satisfaction			Burnout		
	*M*	SD	*Md*	*M*	SD	*Md*	*M*	SD	*Md*
Gender (*n* = 159)									
Male (*n* = 52)	1.56^*^	0.44	1.44	4.18^*^	0.51	4.20	1.99	0.52	1.89
Female (*n* = 107)	1.77^*^	0.57	1.67	4.02^*^	0.43	4.00	2.11	0.49	2.11
Education level (*n* = 156)									
University (*n* = 137)	1.61	0.49	1.56	4.13	0.48	4.10	2.04	0.52	2.00
Non-University (*n* = 19)	1.77	0.58	1.67	3.89	0.54	3.80	2.09	0.47	2.11

^*^p < 0.05 significance.

#### 3.5.7 Mann–Whitney U test between demographics and the moral injury event scale (MIES)

The Mann–Whitney U Test was used to compare differences between demographic questions, which only included two categories: gender and education level groups in relation to the MIES. There were no significant differences in medians related to betrayal and transgressions (*p* > 0.05). Table 4 shows the frequencies and differences of medians for gender and university level in the MIES.

**Table 4:** Frequencies and Means for difference of medians for gender and education level in the Moral Injury Event Scale.

	Betrayal			Transgressions		
	*M*	SD	*Md*	*M*	SD	*Md*
Gender (*n* = 107)						
Male (*n* = 52)	4.45	1.32	4.67	4.43	1.29	4.60
Female (*n* = 107)	4.30	1.48	4.50	4.55	1.39	4.80
Education level (*n* = 156)						
University (*n* = 137)	4.35	1.40	4.67	4.13	0.48	4.1
Non-University (*n* = 19)	4.63	1.05	4.67	3.89	0.54	3.8

^*^p < 0.05 significance.

#### 3.5.8 Kruskal–Wallis analysis

A total of five independent Kruskal–Wallis tests were run to determine differences between groups in variables with more than two categories (age, income, education level, major, time working in the swine industry, time working at the farm, and farm work unit). The results showed no difference in the demographic variable results between compassion satisfaction, burnout, and secondary traumatic stress on the ProQOLS scale and did not show differences on the transgression and betrayal scales on the MIES (*p* > 0.05).

#### 3.5.9 Spearman’s correlations

Spearman’s correlations analysis was run between the survey scales to identify associations and relationships between variables. A significant correlation was found between most variables except for betrayal and satisfaction. The most positive relationship was established between transgression and betrayal (r_s_ = 0.64). Conversely, the highest negative relationship was established between burnout and satisfaction (r_s_ = −0.62). Table 5 shows the matrix of correlations between transgression, betrayal, satisfaction, burnout, and stress. Tables 6 and 7 show the linear regressions for transgressions and stress.

**Table 5:** Matrix of correlations (*n* = 163).

Variable	1	2	3	4	5
1. Transgressions	–				
2. Betrayal	0.64^*^	–			
3. Satisfaction	0.21^*^	−0.014	–		
4. Burnout	−0.37^*^	−0.18^*^	−0.62^*^	–	
5. Stress	−0.39^*^	−0.27^*^	−0.27^*^	0.37^*^	–

^*^p < 0.01.

**Table 6:** Linear regressions for transgressions (*N* = 163).

Variable	Coefficient	Standard Error
Intercept	4.14^*^	1.24
Betrayal	0.52^**^	0.06
Satisfaction	0.02	0.20
Burnout	−0.63^*^	0.21
Stress	−0.49^*^	0.17
$R^{2}$	0.51	

The *t*-statistics are denoted as ^**^p < 0.01 and ^*^p < 0.05.

**Table 7:** Linear regression for stress (*n* = 163).

Variable	Coefficient	Standard error
Intercept	1.82	0.58
Transgressions	−0.11^*^	0.36
Betrayal	−0.02	0.03
Satisfaction	−0.04	0.09
Burnout	0.26^*^	0.10
$R^{2}$	0.26	

The *t*-statistics are denoted as ^**^p < 0.01 and ^*^p < 0.05.

## 4 Discussion

### 4.1 To develop an instrument to identify swine caretakers’ attitudes toward pig euthanasia

This instrument was developed using demographic and swine management questions; in addition, current scales from other fields were adapted for swine industry caretakers. According to Griner and Smith (31), a culturally adapted mental health intervention analysis indicated that experiences of anxiety, depression, work-related stress, and help-seeking behaviors vary across cultures and other demographic variables. The findings of our study have provided valuable insights into the demographic characteristics of Hispanic caretakers which can influence decision-making, having an adverse impact on the timely euthanasia of pigs and, consequently, animal welfare.

In a study by Mullins et al. (12), managers also suggested that cultural factors might contribute to the delay in timely euthanasia. Therefore, in our study, the first two survey sections, demographic characteristics and swine management, aimed to identify caretakers’ attitudes toward pig euthanasia. The Impact of Event Scale did not show any variability in this study, which may have been due to the lack of variability and instrument dependency. In addition, this could have been due to inadequate question design or an incorrect type of question (not interpreted the same in Spanish as in English) or the effects of the context or a combination of all the variables mentioned before these.

A recent study aimed to evaluate whether the attitudes of Mexican TN-Visa swine caretakers differ by gender (female or male). The study surveyed participants through 9 demographic questions and 31 questions adapted from the study by Rault et al. (32), which were categorized by (a) *confidence*, (b) *knowledge*, (c) *decision,* and (d) *comfort*. According to the results, regardless of gender comparisons, the caretakers’ answers indicated that they did not face any difficulty in deciding when to perform euthanasia. This supports our study findings, which showed no gender differences in performing euthanasia and swine management practices. Additionally, the participants indicated that they were confident in identifying illness and health outcomes and knowing when to perform euthanasia (33). This study highlights the growing importance of filling the gap in understanding the impact of euthanasia on swine caretakers, especially in regard to gender differences. Given the complexity of psychological and sociocultural barriers, this exploratory study demonstrated the necessity of proposing other methodologies to understand the context of Hispanic caretakers attitude regarding pig euthanasia.

### 4.2 To assess and describe swine caretakers’ attitudes toward pig euthanasia using the developed surveys

The demographic results showed that 31.9% (*n* = 52) of participants in this study were female. This result shows that a percentage of women participates in the swine industry. Unfortunately, not much has been studied about women’s roles in this field. However, the United States Department of Agriculture (USDA) census showed that the statistics for female agricultural producers in the US between 2012 and 2017 increased by 27%. In addition, the percentage of women in swine farming increased from 9 to 10% of the total swine producers (20). These increases in women in the swine field demonstrate the need for studies to focus on gender influences on swine caretaker practices.

We found that 71.2% (*n* = 116) of the caretakers in our study were 18–35 years of age. Almost all participants in this study were from Mexico, 96.9% (*n* = 158). This could be related to the NAFTA professional (TN) visa requirements, as applicants must be Mexican or Canadian to opt for this work permit. The level of education and major education results must be considered as part of this requirement. The conditions for this visa include that caretakers must have a profession on the NAFTA visa list, and the professional needs qualification and education related to the work field (24). Caretakers with a university education were more likely to identify pigs that need to be euthanized without help; yet, training with a One Welfare approach could be tailored to areas they struggle with, i.e., confidence in their assessment, so they do not have to wait for others to euthanize pigs and mental health to aid in better coping skills associated with the nature of the work.

Although the results of this study found that most workers were under the average income, this was not associated with dissatisfaction. However, lower income may lead to dissatisfaction and could be interesting to evaluate if it affects caretakers’ job satisfaction. A study of pay inequality, job satisfaction, and firm performance (34), analyzing salaries and company reviews, found that pay inequality perceived by employers is strongly associated with job satisfaction; however, they found it hard to identify a damage to morale related to the base pay. Moreover, monetary incentives could be related to differences in productivity.

The results associated with time working on the farm and the swine industry were similar. Thus, many participants in this study started working in the swine industry as their first farm job. The farm work unit participants’ responses varied in knowledge of the Common Swine Industry Audit (CSIA; *p* = 0.005). Participants’ results showed that 53.47% of the farrowing unit caretakers were unaware of the CSIA, while 26.83% of the breeding unit lacked awareness of this audit. Similarly, 27.78% of participants who worked in both units were familiar with the CSIA. These meaningful results may be due to the different tasks caretakers perform during their daily inspections of barns. Each farm work unit is diverse in techniques that caretakers must conduct to achieve their particular unit production goals (Rea, 2018).

In a study by Mullins et al. (12), 72 participants, representing 44.17% of the sample, showed a lack of awareness of the CSIA. The deficiency in awareness about these guidelines/standards is concerning, since failure to euthanize injured pigs may lead to increased animal suffering, compromised animal welfare, and financial losses. The present study found that as experience and time working on the farm increased, so did caretaker awareness of the CSIA (*p* < 0.001). Our results suggest that education and training should be strengthened around swine industry standards to ensure timely and humane pig euthanasia when specific conditions are met. The CSIA standards are critical guidelines to ensure animal welfare. Although the CSIA guidelines are clear on what animals should be euthanized immediately, it seems that new workers still struggle to identify these conditions. This finding is directly related to our results, showing that new workers do not know the CSIA standards. One must understand that new workers are not necessarily exposed to the CSIA standards *per se*, but they are trained on the standards. Our results suggest a need to strengthen education and training around swine industry standards to ensure timely and humane pig euthanasia when specific conditions are met. The CSIA standards are critical guidelines to ensure animal welfare.

Our results indicate that as caretaker gain experience/time working at the farm increases, so does caretakers’ confidence in their ability to identify compromised pigs and conduct pig euthanasia (*p* < 0.001). More than half (52.27%) of the caretakers with less than a year of experience were undecided about when a pig needs to be euthanized and preferred not to perform euthanasia themselves. On the other hand, more than three-quarters (78.79%) of caretakers with more than 3 years of experience felt capable of identifying injured pigs and performing euthanasia themselves. Considering these results, one of the reasons for the inexperienced caretakers’ unwillingness to euthanize pigs may be their lack of confidence in competently identifying compromised animal/s and their ability to proficiently/effectively euthanize the animal/s. Similarly, other authors have reported that the more time employees perform pig euthanasia, the more willing they are to conduct this practice (35). These findings could be used to develop further training for more experienced staff to mentor junior staff, with the aim of reducing the time it takes for newer staff members to become confident in identifying compromised pigs and proficiency in performing euthanasia.

Furthermore, a recent study of animal caretakers’ perspectives on performing euthanasia on commercial sow farms indicated that euthanasia becomes easier the more times it is performed, relating this to the skills that caretakers obtain with experience (36). At the same time, investigation that focused on Austrian veterinarians’ attitudes toward euthanasia showed that veterinarians who have worked for few years performing this process were more likely to disagree with it in some convenience scenarios (13). The findings of the previous research and this study support the perception that caretakers’ lack of experience may negatively affect their preferences in performing euthanasia, as they may associate euthanasia as being a negative thing (as a life must be ended), but in reality, it is a positive thing (as it prevents animal suffering).

### 4.3 To identify the demographic and psychological barriers associated with timely pig euthanasia performance

Secondary traumatic stress and transgression were significantly correlated scales associated with burnout, betrayal, and worker satisfaction. Furthermore, individuals identifying as female had higher secondary traumatic stress scores and lower compassion satisfaction scores. This data suggest that there are demographic, psychometric, and training-related factors correlated with Hispanic caretakers’ feelings about euthanasia. The results from the MIES did not show differences between perceived betrayal and transgression feelings. This scale has been previously used with veterans by Norman and Maguen (37) and helped to understand morally injurious events in the context of war, such as killing or harming others. Some of the recommendations for this study were to apply an empirical evaluation of the model, do longitudinal studies of course and associated factors, and distinguish between witnessing, perpetration, and betrayal. Norman and Maguen (37) also concluded that moral injury can lead to post-traumatic stress disorders, depression, and other disorders in which feelings such as guilt, shame, betrayal, and anger are predominant, although these feelings may occur in the absence of a formal disorder (37). The surveys used in this study have not been previously used on pig caretakers but have been utilized with shelter animal caretakers. The MIES has been used with shelter animal caretakers (29), as shelter animal caretakers may experience direct trauma when they have to euthanize healthy animals. They can experience meaningful events such as public scorn and harassment, which could also cause direct trauma. In a study by Wisco et al. (38) about moral injury in US combat, veterans reported that 25.5% endorsed transgression by others and 25.5% endorsed betrayal, these were assessed using the MIES. A recent study reported experiences and the impact of moral injury in veterinary professionals in the United Kingdom (39) using a modified version of the MIES and highly associated symptoms about post-traumatic stress disorders with experiences of moral injury. These findings are consistent with our study. Finally, although this study focused on Hispanic caretakers, the psychological effects of euthanasia can possibly be extrapolated to other sectors/populations.

The adaptation of these scales has helped us compare and understand how scales related to feelings and experiences in other fields and languages can also be used on-farm for caretakers who perform pig euthanasia. Multilingual social workers assisting Latinos with trauma therapy in Spanish (40) acknowledged that language works on levels of associations, meanings are hard to translate, and that different languages hold different worldviews. It is possible that the wording of the questions could have a different context than the caretakers’ native language and cultural experience of moral injury or traumatic stress.

#### 4.3.1 Gender differences

We found that there was no difference between the perspectives of male and female participants concerning euthanasia. However, gender results showed a difference in secondary traumatic stress (*p* = 0.03) and compassion satisfaction (*p* = 0.02) between male and female caretakers performing pig euthanasia. Yarian (41) evaluated if caretaker attitudes toward euthanasia differed between women and men, but differences in confidence, comfort, knowledge, and decision-making attribute factors were not reported. However, Mullins et al. (12) who explored caretaker decision-making determined that female employees showed more negative attitudes toward pig euthanasia. Our results on the ProQOLS support significant gender differences when performing pig euthanasia and higher secondary traumatic stress and lower compassion satisfaction in women than men. These results may be due to a difference in the gender roles at the swine farm. These findings may also indicate that females are impacted differently than males when performing pig euthanasia and this work may relate to gender inequality, therefore, complicating females’ euthanasia decisions, performance, and responsibilities.

In a study comparing the relationship between caretakers and animals in the swine industry, Porcher (42) mentioned that women are forced to repress their spontaneous affection toward the animals. A study that evaluated mental health among Latina farmworkers and other employed Latinas showed that female farmworkers, compared with non-farmworkers and unemployed women, had higher stress and anxiety (43). These results indicate a problem inside the agricultural industry and are essential factors that should be considered for reducing gender inequality in the swine industry. The authors also highlighted the critical aspect of work–family balance, which disproportionately affects women’s work and personal stress levels. For instance, a study about stress and depression among Latina women in rural areas in North Carolina identified the Latina population’s mental health as poor, where important levels of depression and stress presented significant risks to their health (44). Some of the most critical stressors affecting their lifestyle include marital status, lack of finances, language barriers, and difficulty being away from family members. Connecting to our study, women’s stress and job satisfaction may be affected by external events in their daily personal life. The balance between home/work responsibilities of the Hispanic woman are factors that should be highly considered as possible cultural barriers that may indirectly impact timely euthanasia, as stress and burnout may already be high.

#### 4.3.2 Correlations between stress and the moral injury event scale (MIES)

In this study, The Professional Quality of Life Scale (ProQOLS) and Moral Injury Event Scale (MIES) results have a significant correlation. Secondary traumatic stress was negatively correlated with the MIES (betrayal and transgression). In a study on compassion fatigue in animal shelter employees, MIES was also negatively correlated with secondary traumatic stress; thus, the trauma experienced by animal care employees may be due to moral stress (29). A high MIES score is indicative of lower moral injury, but this may also imply that Hispanic caretakers in this study were affected by moral stress associated with performing pig euthanasia, performing tasks such as hospital pen checking, telling others when to euthanize a pig, and present and past job experiences.

## 5 Conclusion

This study utilized a compilation of demographic questions, swine management surveys, and validated psychometric measures previously used to measure occupational stress in a variety of caring professions, to explore euthanasia-related decisions in swine farm personnel. Additionally, the study tackled the critical issue of understanding the underlying cultural barriers that prevent the Hispanic workforce from conducting timely euthanasia to prevent animal suffering and improve animal welfare on-farm. Not only should the agricultural industry seek to care for the welfare of its animals but also to understand the workforce that ultimately helps feed America. Our findings show that training on-farm needs to significantly improve and cater to the level of education of the worker to be most effective. It was clear in this study that the level of education of caretakers was higher due to the use of caretakers hired with NAFTA visas. This added another level of complexity to this study, as most US residents/citizens who work as caretakers on-farm generally do not have a college degree. Thus, the needs of caretakers with NAFTA visas may vary from caretakers without a high level of education. Furthermore, this study indicates that we may need to utilize people to euthanize that are less vulnerable/susceptible to stress; overall, we need to instill trust in the caretakers who have been trained well enough to successfully/proficiently complete euthanasia. In addition, training caretakers to identify and perform euthanasia confidently and competently and further focusing on personnel with difficulty making euthanasia decisions and those individuals who are less experienced may improve participation and understanding of effective euthanasia practices. Our results show that caretakers are not 100% comfortable identifying sick/compromised animals (if they are trained properly, anyone who works with animals should know when an animal is sick and requires euthanasia according to the CSIA guidelines). This multi-disciplinary study was designed to identify the barriers associated with pig timely euthanasia knowing that there would be some mental health related implications, but we also found clear gender differences that the swine industry should account for. It is also important to establish a framework for strategies based on gender inequality, providing welfare to the animal but considering caretakers’ mental health and training for women caretakers performing euthanasia.

## 6 Limitations and recommendations for future studies

This study was exploratory, whereby the results cannot be extrapolated to the swine industry. It is recommended that future studies on swine caretakers evaluate if the internal consistency of surveys is acceptable for the measures to be evaluated. In this way, the ProQOLS and the MIES could be considered instruments to evaluate swine industry conditions and help to find solutions for this field. Future studies should also develop focus groups based on the results obtained from the quantitative analysis, to better understand the underlying impact of euthanasia on swine caretakers that the surveys may not have identified.

The IES-R was not useful for surveying swine caretakers in this study, and we did not find gender or age-related differences in the Swine Management. Future projects should further evaluate if this scale is appropriate for the swine industry or consider alternative methodologies to measure the variables related to this scale and develop different questions to assess the Swine Management Section results to identify gender and age group differences associated to euthanasia practices.

Education level should be considered when developing trainings, as many of the caretakers in this study had advanced degrees and others did not. Furthermore, the farm work unit (phase of production the workers are responsible for) should be looked at individually to identify weaknesses associated to the specific work unit. It is clear in this work that knowledge of the CSIA varied based on the work unit, leading us to speculate that the farms could benefit from a mentorship program to standardize knowledge and practices throughout the company. Improving worker confidence in performing euthanasia could include practicing on cadavers and continuous mentorship. Further, focusing on personnel with difficulty making euthanasia decisions and those with less experience could improve timely euthanasia practices.

Establish a framework on strategies based on gender differences and inequality proving welfare to the animal but considering mental health and training for caretakers performing euthanasia could be beneficial. Stress and transgression were identified as the most robust correlated variables to burnout, betrayal, and work satisfaction. Therefore, future on-farm strategies should focus on the factors affecting caretakers’ psychological assistance, therapy, or training to manage emotions. Swine farms should look for ways to help understand how these feelings affect caretakers’ daily life and labor life to provide skills that may reduce these effects on pig caretakers. Furthermore, not explored by the study, the task of euthanizing pigs is a normal expectation of most farm caretaker positions. Stress and transgressions were identified as the most robust correlated variables to burnout, betrayal, and work satisfaction. Therefore, future on-farm strategies should focus on providing psychological assistance, therapy, or training to manage emotions. Establishing a framework based on gender differences and equality while considering mental health and training for caretakers performing euthanasia could be highly beneficial. Furthermore, not explored by the study, the task of euthanizing pigs is a normal expectation of most farm caretaker positions. Therefore, during recruitment and hiring managing expectations of what the role entitles should be clear in addition to proper communication upon hiring (e.g. good and clear communication channels, dignity at work policies). Undoubtably, euthanizing animals could result in extreme stress, particularly if euthanasia is associated to poor husbandry practices.

We recognize that a limitation of this study is how we collected the data. Since the participants were sitting next to each other at a large table, they may have experienced pressured to answer fast (not going in depth about certain things) and fear of others looking at their answers. For future studies we would recommend placing the participants in a large room to allow enough space between each participant, in order to prevent them from seeing other participant’s answers and talking to each other. Therefore, future studies should evaluate the best data collection method.

Finally, the Hispanic workforce is diverse. However, we found that most participants were Mexican. Therefore, our findings are not representative of the entire swine industry in the US. These farms just happened to have a large Mexican population, but there can be variation across the US. We also did not expect to find such a high level of education. However, given that many of the workers came to the US with NAFTA visas, it is required that they have a high level of education to be given a visa to work in the US. The high level of education impacted our results that people who are educated can experience a different kind/level of frustration (such as management strategies, equipment failure, the demand to meet production goals, and other reasons; Garcia et al., unpublished data). The increase in NAFTA visas is changing the agricultural workforce, and therefore, we must adopt different training and continuing education strategies to ensure we are addressing the needs of a workforce that varies in culture and education.

## Data availability statement

The original contributions presented in the study are included in the article/Supplementary material, further inquiries can be directed to the corresponding author.

## Ethics statement

The studies involving humans were approved by The Human Research Protection Program (IRB 2019-225). The studies were conducted in accordance with the local legislation and institutional requirements. The participants provided their written informed consent to participate in this study.

## Author contributions

NA: Formal analysis, Investigation, Writing – original draft, Data curation, Software. PJ: Data curation, Investigation, Software, Writing – original draft, Writing – review & editing, Formal analysis, Methodology, Validation, Visualization. CG: Investigation, Data curation, Formal analysis, Methodology, Software, Writing – original draft. AB-A: Validation, Supervision, Writing – original draft. AA: Writing – review & editing. MS: Writing – review & editing. JM: Writing – review & editing. AG: Conceptualization, Funding acquisition, Methodology, Investigation, Project administration, Supervision, Resources, Writing – review & editing, Visualization, Data curation, Validation.
