# Supplementary material for: Hispanic worker attitudes toward pig euthanasia on U.S. farms
Source: Front Vet Sci. 2024 Apr 2;11:1281102. doi: 10.3389/fvets.2024.1281102 (PMC11019433; doi:10.3389/fvets.2024.1281102)

Supplementary Material

Supplementary Figure 1: Distribution of Participants by Gender

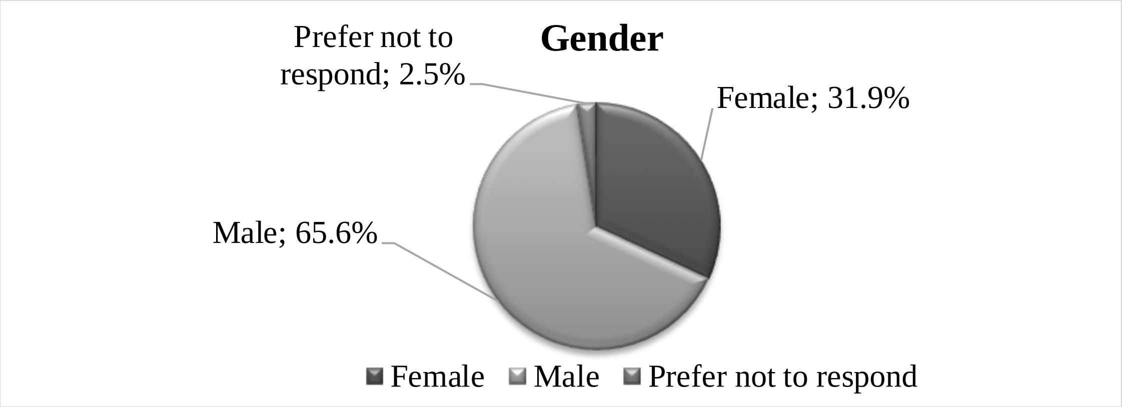

Supplementary Figure 2: Distribution of Participants by Country

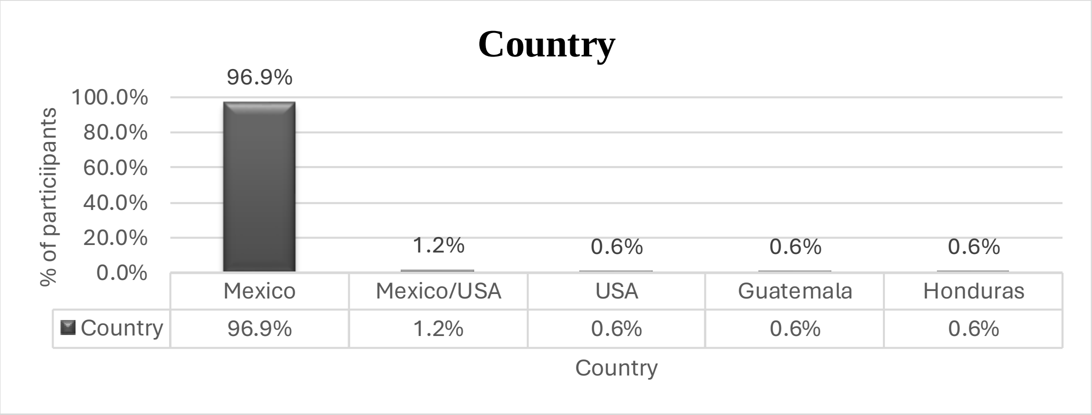

Supplement: Supplementary file 1 [file Image_1.pdf]
